# The causal impact of smoking behavior on osteoarthritis: a Mendelian randomization analysis

**DOI:** 10.3389/fpubh.2025.1437443

**Published:** 2025-01-23

**Authors:** Qiang Xiao, Susu Dong, Yafen Tan, Xuan Zhang, Lu Yao, Qiuping Li, Tianli Wang

**Affiliations:** Department of Pulmonary and Critical Care Medicine, Changde Hospital, Xiangya School of Medicine, Central South University (The First People’s Hospital of Changde City), Changde, Hunan, China

**Keywords:** genetic association, Mendelian randomization, meta-analysis, osteoarthritis, smoking

## Abstract

**Objective:**

Although smoking and osteoarthritis (OA) have been linked in a number of studies, the exact cause of the association is still unknown and the conclusion is controversial. The purpose of this study was to use Mendelian randomization (MR) analysis to investigate the causal relationship between smoking phenotypes and OA risk from a genetically informed standpoint.

**Methods:**

As instrumental variables (IVs) based on single nucleotide polymorphisms (SNPs), this study used the summary-level data of corresponding genome-wide association study (GWAS) for five smoking phenotypes involving 1,694,781 samples. The outcomes comprised both a discovery and a replication cohort. The discovery MR analysis involved 12 OA traits (177,517 cases and 649,173 controls) while the replication MR analysis incorporated an additional OA GWAS dataset consisting of 36,185 cases and 135,185 controls. The main analytic approach we used was the inverse variance weighted (IVW) method. MR Egger, Weighted median, Weighted mode, and Simple mode were among the other methods that were tested. We conducted meta-analysis to combine the MR results. To confirm the robustness of the results, sensitivity analysis using Leave-One-Out (LOO), level pleiotropy testing (MR Egger intercept test and MR-PRESSO), and heterogeneity testing were performed.

**Results:**

Summary-level MR analysis revealed a positive correlation between genetic predisposition for smoking and the likelihood of developing OA. The meta-analysis merge showed that smoking initiation increased the risk of knee OA by 20%, hip OA by 16%, and knee/hip OA by 19% (all *p* < 0.001). Similarly, lifetime smoking elevated the risk of knee OA by 101%, hip OA by 55%, and knee/hip OA by 84% (all *p* < 0.001). The sensitivity analysis’s findings reinforced the reliability of these findings.

**Conclusion:**

According to our research, smoking increases the likelihood of developing OA from a genetic standpoint. Reducing tobacco use could, therefore, be beneficial in lowering the incidence of OA.

## Introduction

Over 300 million individuals globally suffer from osteoarthritis (OA), the most prevalent kind of degenerative musculoskeletal disorder, particularly the older adult population (1). Osteochondral degradation is a characteristic of OA, which has a substantial negative influence on patient’s quality of life and social functioning and presents as chronic joint pain, stiffness, edema, and decreased mobility (2). A major burden on public health and healthcare economics is the growing incidence of OA, which is linked to demographic aging and rising obesity rates (3). The hip, knee, and spine are the main weight-bearing joints that are impacted. There have been findings linking the risk of OA to characteristics such age, gender, and obesity (4). Although factors involving genetic variation, mechanical injury, immunological inflammatory response and metabolic abnormalities were suggested, the underlying process of OA have not yet been fully understood (5, 6). Since the cause of OA is unknown, there is no proven treatment. Commonly used therapies for OA today include non-steroidal anti-inflammatory medication for pain management, joint cartilage repair agents, and general physical therapy to slow down the disease’s progression. Nevertheless, these therapeutic approaches frequently have less than ideal outcomes (7). Thus, identification of modifiable risk factors to mitigate its impact on individuals and society is crucial. Given the lack of a definitive cure for OA, understanding its pathogenesis and developing effective preventive measures are critical areas of focus for future research and clinical intervention.

There was conflicting evidence in earlier research on the link between smoking and the risk of OA. Several studies have indicated that smoking may have a preventive effect on OA. According to a cross-sectional investigation, smoking is negatively correlated with the prevalence of knee OA in the senior Korean population as a whole (8). A comprehensive review and meta-analysis of 34 independent observational studies conducted in 2017 found that smoking cigarettes was associated with a lower risk of developing knee OA, regardless of study type, and that this link was stronger in males (9). On the other hand, smoking was positively associated with the prevalence of OA in the general US population, according to a cross-sectional study that used the National Health and Nutrition Examination Survey database (10). Meanwhile, a Baseline Cross-Sectional and Longitudinal Multicohort Study found no conclusive link between smoking and the incidence, prevalence, or development of radiographic or symptomatic hip OA at baseline or during a follow-up period of 4–5 years (11). In general, smoking may have an effect on the onset of OA, however there is ongoing debate over the precise causative relationship and relative significance. It is crucial to remember that a large portion of this evidence comes from conventional observational studies, which are prone to measurement error, confounding, and reverse causality. Furthermore, randomized controlled trials (RCT) are not practical for addressing this issue because forced exposure to cigarettes is unethical.

Mendelian randomization (MR) analysis reduces confounding and reverse causation biases by simulating a RCT and evaluating causal links between exposures and outcomes using genetic variants as instrumental variables (IVs) (12). This approach provides a useful substitute for RCTs and has been extensively used in genetic epidemiology to more precisely and reliably examine causal links between risk variables and diseases using data from genome-wide association study (GWAS). By successfully reducing confounding variables and reversing causality biases, MR offers a reliable method for examining the causal relationships between modifiable exposures and disease outcomes as well as producing clinical evidence. Currently, there are several MR studies on smoking and OA; however, the conclusions have been inconsistent due to various factors such as limited instrumental variables and outcome sample sizes, among others. For instance, Ni et al.’s (13) MR analysis revealed that smoking initiation was linked to an increased risk of OA whereas Lee (14) and Johnsen et al. (15) discovered that smoking habit was linked to a lower risk of OA. Thus, using the most complete set of data available, we conducted a two-sample MR analysis based on publicly available GWAS data to investigate the possible causal relationship between cigarette smoking and the onset of OA. The findings offer insights with substantial relevance to clinical practice and public health.

## Methods

### Study design

**Figure 1:**
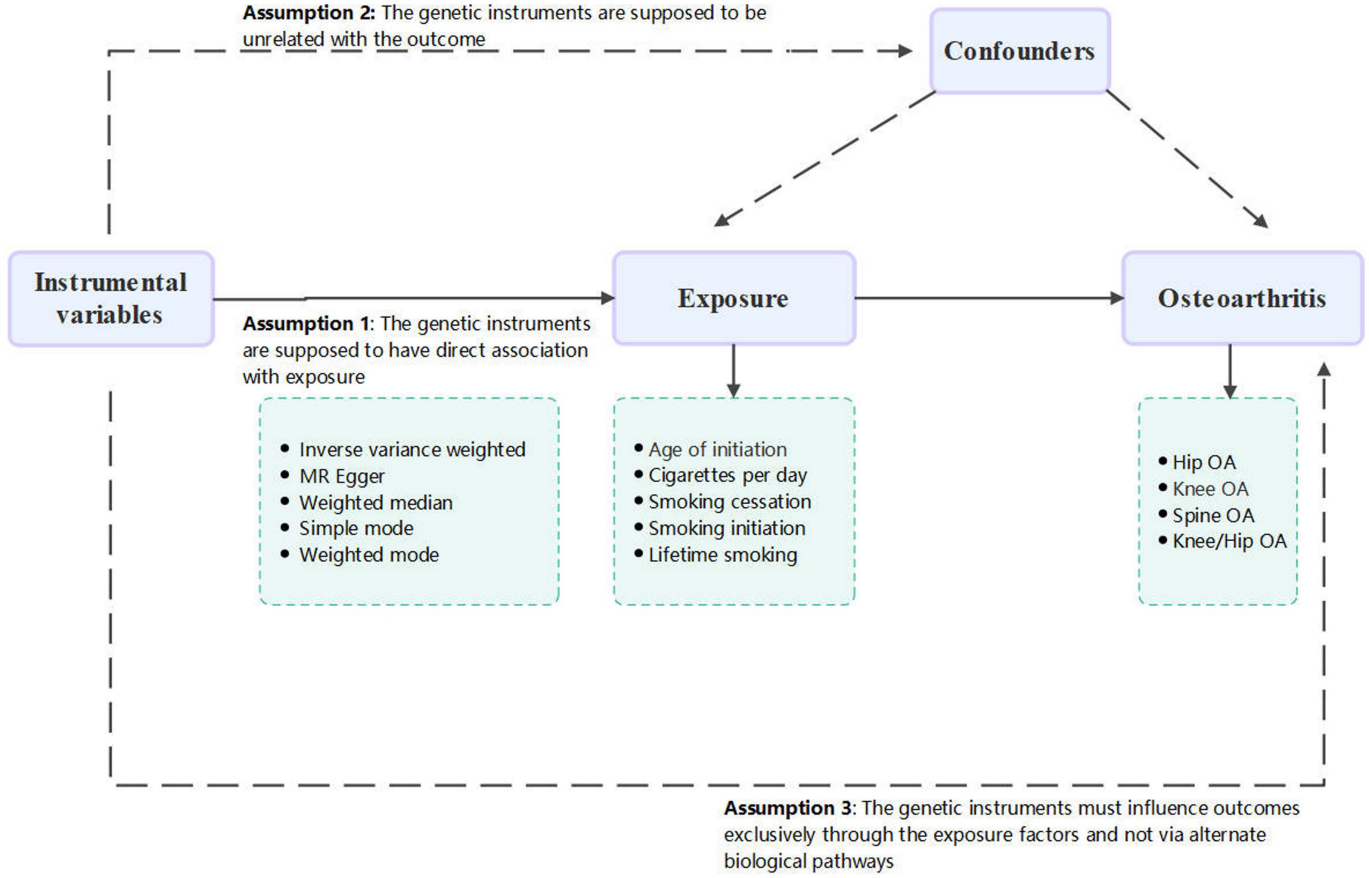
Flow chart of the MR study.

This study conducted a thorough and in-depth two sample MR analysis in order to investigate a possible causative link between smoking and OA. There was no need for further ethical approval or consent to participate because this study is based on publicly available databases and published works. A convincing MR analysis must satisfy three fundamental assumptions in order to establish a causal estimate: (1) the genetic instruments must be directly associated with exposure (in this case, smoking behavior), and this was considered attained as the SNPs were identified from the large-scale GWAS meta-analysis; (2) the genetic instruments must be unrelated to the outcome (in this case, OA) and unaffected by any identified or unidentified confounding factors; and (3) the genetic instruments must only affect outcomes through the exposure factors and not through other biological pathways. The conduct and interpretation of our MR study comply with the STROBE-MR (Strengthening the Reporting of Observational Studies in Epidemiology – Mendelian Randomization) guidelines, ensuring robust and transparent reporting of our methodology and findings (16). The study’s summary is shown in Figure 1.

### Data source for smoking behaviors

To obtain the smoking genome-wide association summary dataset, the GWAS and Sequencing Consortium of Alcohol and Nicotine use (GSCAN) used the most recent GWAS data, which included 1.2 million people with European ancestry (17). Twenty-nine studies in all were included in the GWAS data, with 23andMe, UK Biobank, deCODE, and HUNT being major contributors since they provided a significant number of samples. There were four types of smoking phenotypes: smoking initiation (a continuous phenotype, age of initiation of regular smoking), smoking cessation (a binary phenotype, contrasting current versus former smoker), smoking initiation (a binary phenotype, ever versus never being a regular smoker), and cigarettes per day (a continuous indicator of smoking heaviness). Additionally, lifetime smoking was incorporated as a complete phenotype utilizing data from the UK Biobank, which recruited 462,690 individuals with a preponderance of European ancestry (18). The lifetime smoking measure is a continuous indicator that combines the burden of lifetime smoking exposure, which is based on smoking initiation, duration, heaviness, and cessation (Table 1).

**Table 1:** Characteristic of data in this study.

Trait		Sample size	Population	Data source
OA in discovery source			Asia, Europe, and European Americans	Boer’s lab; https://msk.hugeamp.org; (PMID:34450027)
	Hip OA	353,388		
	Knee OA	396,054		
	Spine OA	333,950		
	Knee/Hip OA	490,345		
OA in replicate source			European	UK Biobank; https://ega-archive.org; (PMID:29559693)
	Hip OA	11,989		
	Knee OA	22,347		
	Knee/Hip OA	32,970		
Smoking phenotypes				
	Age of initiation	341,427	European	GSCAN consortium; https://doi.org/10.13020/3b1n-ff32; (PMID:30643251)
	Cigarettes per day	337,334		
	Smoking cessation	547,219		
	Smoking initiation	1,232,091		
	Lifetime smoking	462,690	European	UK Biobank; https://doi.org/10.5523/bris.10i96zb8gm0j81yz0q6ztei23d; (PMID: 31689377)

### Data sources for OA

Two separate GWAS provided the outcome data for OA. The most extensive GWAS database currently in use provided the primary data for OA. It contained 12 OA-related features that were aggregated from 9 worldwide cohorts, totaling 826,690 people from Asia, Europe, and European Americans, including 177,517 OA patients (1). A supplemental dataset of 181,370 people (36,185 with OA) was obtained from the GWAS database, including five OA characteristics, for the replication of MR analysis in order to support the validity of the study results and reduce the possibility of false positives (19). These original papers provide further details.

### Selection of the genetic instruments

To guarantee the accuracy and robustness of causal inferences, the choice of IVs is essential. All SNPs with genome-wide significance (*p* < 5E-08) that independently and strongly predicted exposures were used. The linkage disequilibrium (LD) structure of European populations was used to determine which independent SNPs [clumping distance (kb) = 10,000, *r*^2^ < 0.001] were kept from this collection. The chosen instrument SNPs did not include palindromic SNPs with intermediate allele frequencies. Finally, using the formula of $\frac{\left(n-k-1\right)}{k}$ × $\frac{R^{2}}{1-R^{2}}$ , where *n* is the sample size, *k* is the number of SNPs used as instruments, and *R*^2^ is the percentage of variance that the genetic tools explained, the *F*-statistic was computed to quantitatively confirm whether the chosen SNPs were strong instruments. To reduce the chance of mild instrument bias, we made sure that all of the instruments used in the MR analysis had F-statistics greater than 10 (20).

### Statistical analysis

The causal relationships between smoking phenotypes and OA were assessed using the two-sample MR study. As the main, more accurate, and objective approach, multiplicative random-effects inverse variance weighting (IVW) was employed for the causal estimations. Additionally, to exclude for relevant variables, MR Egger, weighted median, simple mode, and weighted mode analysis were used (21). False positives in multiple testing were controlled by using the False Discovery Rate (FDR) adjustment. If the estimated causal impact of a particular smoking phenotype had an FDR <0.05, the connection was considered statistically significant. To make sure the basic causal estimates were robust, many sensitivity analyses were carried out. Heterogeneity between individual genetic variations was evaluated using Cochran’s *Q* statistic, which might suggest the existence of faulty instruments. Horizontal pleiotropy was detected utilizing MR-PRESSO. Outliers that were found were eliminated for a subsequent MR reanalysis. Furthermore, the existence of horizontal pleiotropy was assessed using the MR-Egger intercept and pleiotropy tests; data with a significance level of *p* > 0.05 were considered to be more reliable. Additionally, each instrumental variable was eliminated one at a time in a leave-one-out analysis to see whether any one SNP had an excessive impact on the outcomes. All MR studies were carried out using the “TwoSampleMR” and “MR-PRESSO” software packages in R software (version 4.3.2).

## Results

### Selection of IVs

In the discovery and replication cohorts, 249 and 255 SNPs, respectively, were utilized for the MR analysis after the genetic instrument selection processes and harmonization with the outcome data (see Supplementary Tables S1, S5). From 29.79 to 952.99, the F-statistics were all more than the empirical cutoff of 10 to reduce the weak instrument bias.

### Discovery on the risk of smoking to OA

**Figure 2:**
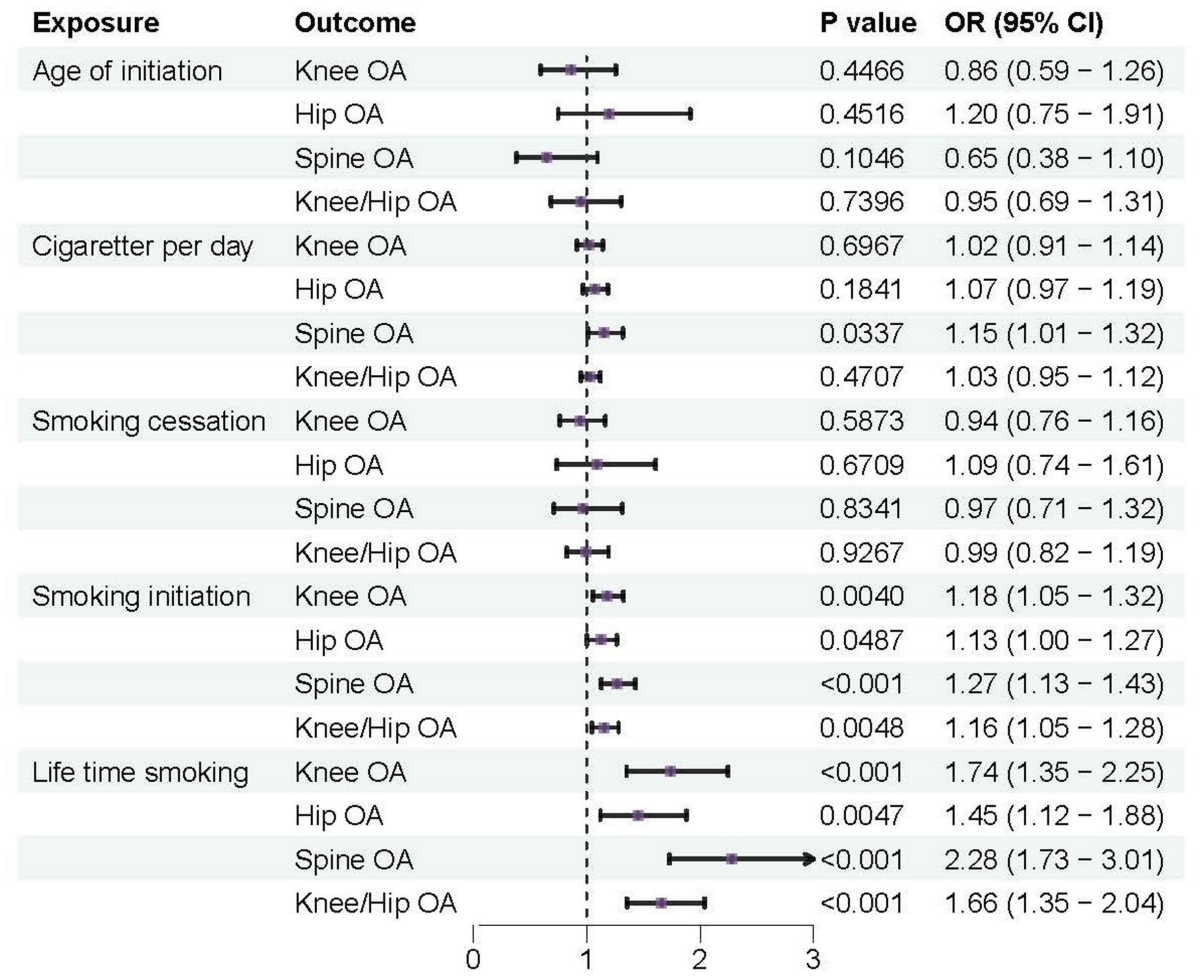
Forest plots of MR analysis by IVW for genetically predicted smoking phenotypes and risk of OA (discovery).

**Figure 3:**
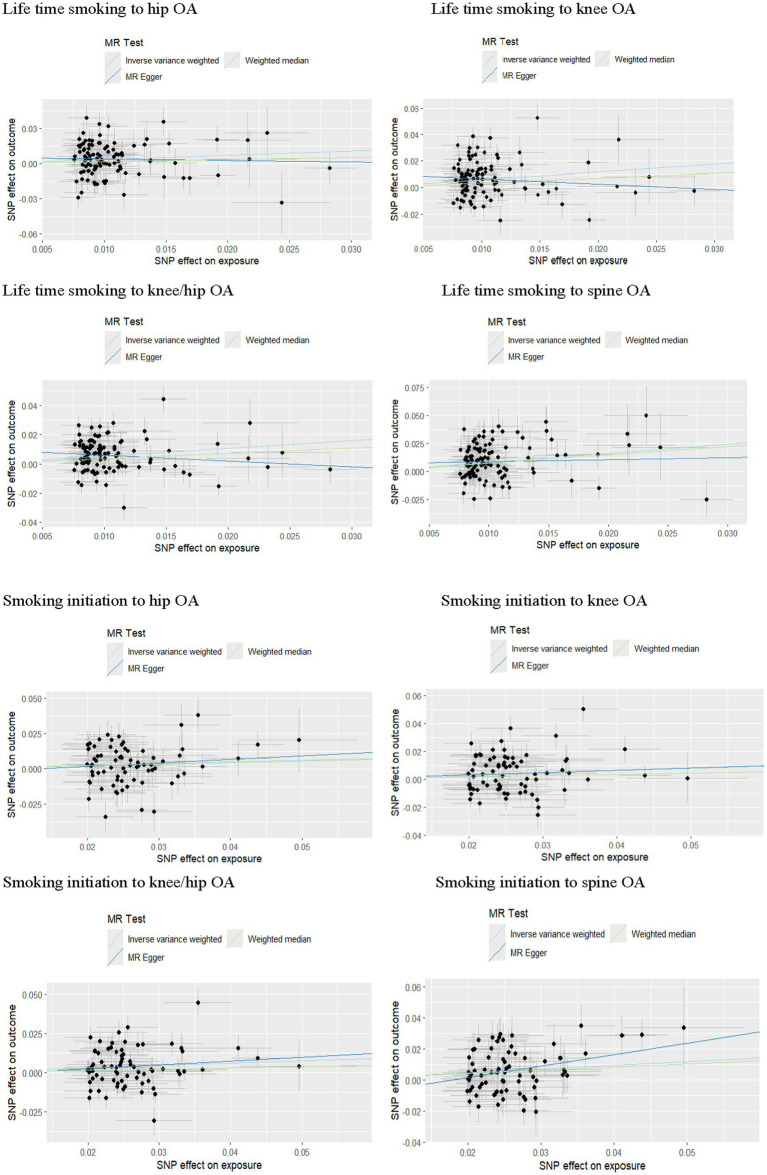
Forest plots of MR analysis by IVW for genetically predicted smoking phenotypes and risk of OA (replication).

According to the IVW analysis, a genetic predisposition to smoking initiation increased the risk of developing knee OA with an odds ratio (OR) of 1.18 (95% confidence interval [CI] = 1.05–1.32; false discovery rate [FDR] = 0.016; *p* = 0.0040), spine OA (OR = 1.27, CI = 1.13–1.43, FDR < 0.001, *p* < 0.001), knee/hip OA (OR = 1.16, CI = 1.05–1.28, FDR = 0.0137, *p* = 0.0048), and the potential risk of hip OA (OR = 1.13, CI = 1.00–1.27, FDR = 0.1081, *p* = 0.0487). Furthermore, a higher risk of knee OA (OR = 1.74, CI = 1.35–2.25, FDR < 0.001, *p* < 0.001), hip OA (OR = 1.45, CI = 1.12–1.88, FDR = 0.0157, *p* = 0.0047), spine OA (OR = 2.28, CI = 1.73–3.01, FDR < 0.001, *p* < 0.001), and knee/hip OA (OR = 1.66, CI = 1.35–2.04, FDR < 0.001, *p* < 0.001) were also substantially linked to increased genetically predicted lifetime smoking (Table 2 and Figures 2, 3). Age of initiation, cigarette per day, smoking cessation did not show a similar association with OA (Supplementary Table S2).

**Table 2:** Mendelian randomization (MR) results of causal links between smoking phenotypes and different OA phenotypes by using IVW method in discovery cohort.

Exposure	Outcome	OR (95% CI)	SE	*P*_IVW_	*P*_FDR_	Heterogeneity *P* for Cochran’s *Q*	*P* for MR-PRESSO	Horizontal pleiotropy *P* for Egger intercept
Age of initiation	Knee OA	0.86 (0.59–1.26)	0.1913	0.4466	0.7443	0.8995	0.714	0.5184
	Hip OA	1.20 (0.75–1.91)	0.2393	0.4516	0.6948	0.7986	0.951	0.8068
	Spine OA	0.65 (0.38–1.10)	0.2700	0.1046	0.2093	0.9719	0.663	0.6327
	Knee/Hip OA	0.95 (0.69–1.31)	0.1643	0.7396	0.8218	0.7059	0.608	0.3440
Cigarette per day	Knee OA	1.02 (0.91–1.14)	0.0566	0.6967	0.8196	<0.001	0.409	0.0043
	Hip OA	1.07 (0.97–1.19)	0.0523	0.1841	0.3348	0.0029	0.209	0.0077
	Spine OA	1.15 (1.01–1.32)	0.0678	0.0337	0.0843	0.0001	0.059	0.0015
	Knee/Hip OA	1.03 (0.95–1.12)	0.0419	0.4707	0.6724	<0.001	0.263	0.0007
Smoking cessation	Knee OA	0.94 (0.76–1.16)	0.1075	0.5873	0.783	0.1015	0.114	0.3342
	Hip OA	1.09 (0.74–1.61)	0.1992	0.6709	0.8386	0.0015	0.688	0.5719
	Spine OA	0.97 (0.71–1.32)	0.1567	0.8341	0.878	0.0843	0.056	0.4123
	Knee/Hip OA	0.99 (0.82–1.19)	0.0940	0.9267	0.9267	0.0868	0.051	0.9729
Smoking initiation	Knee OA	1.18 (1.05–1.32)	0.0581	0.0040	0.016	<0.001	0.418	0.9317
	Hip OA	1.13 (1.00–1.27)	0.0598	0.0487	0.1081	<0.001	0.572	0.7011
	Spine OA	1.27 (1.13–1.43)	0.0604	<0.001	<0.001	0.0142	0.481	0.1019
	Knee/Hip OA	1.16 (1.05–1.28)	0.0518	0.0048	0.0137	<0.001	0.654	0.7662
Life time smoking	Knee OA	1.74 (1.35–2.25)	0.1296	<0.001	<0.001	<0.001	0.495	0.0848
	Hip OA	1.45 (1.12–1.88)	0.1320	0.0047	0.0157	<0.001	0.801	0.3038
	Spine OA	2.28 (1.73–3.01)	0.1408	<0.001	<0.001	<0.001	0.848	0.2183
	Knee/Hip OA	1.66 (1.35–2.04)	0.1043	<0.001	<0.001	<0.001	0.856	0.0349

All statistical tests were two-sided.

**Figure 4:**
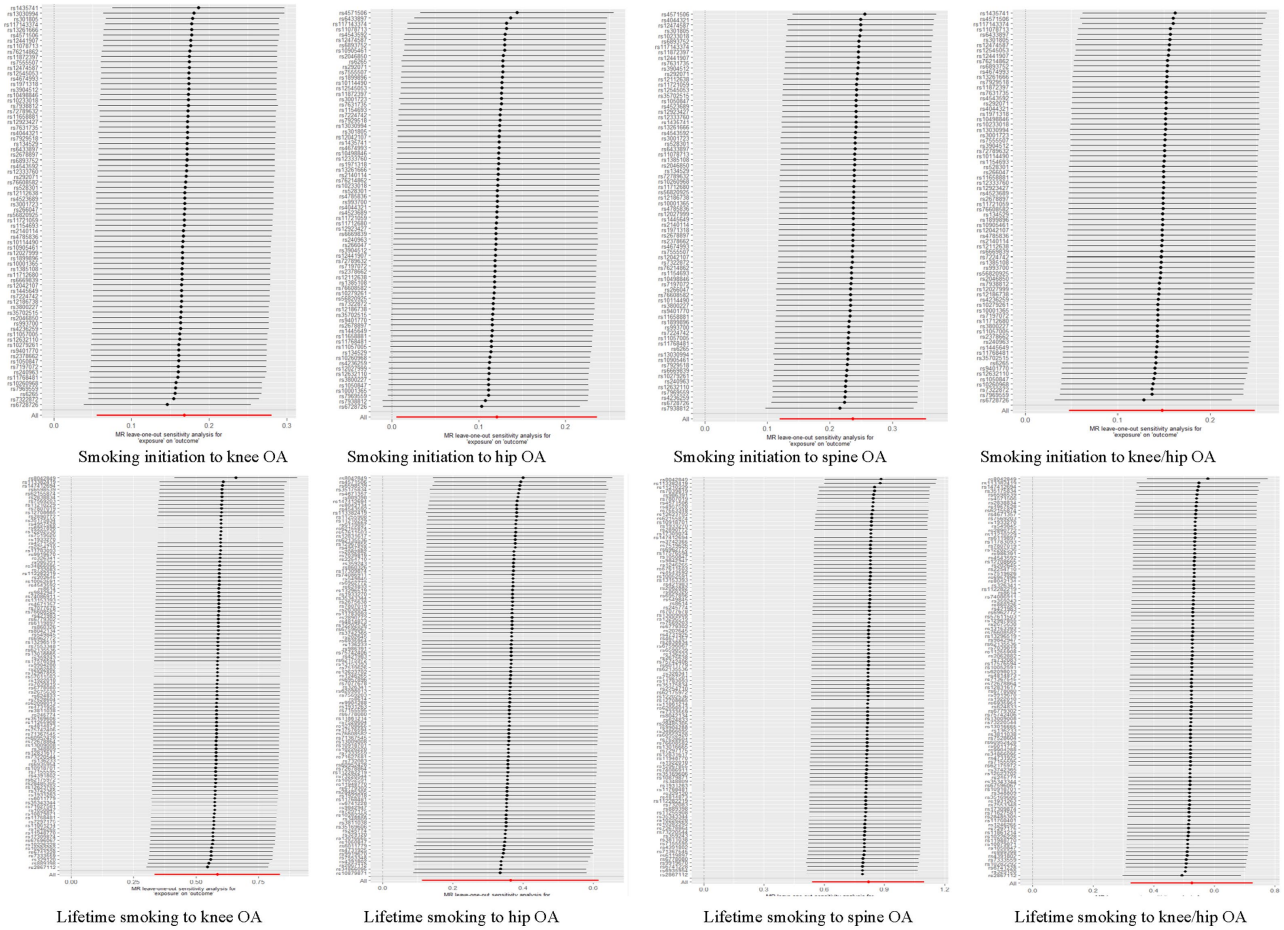
Forest plots of combined results of OA risk from the meta-analysis.

In the sensitivity analysis, we discovered indications of heterogeneity in the MR analysis about the causal relationship between smoking and the risk of OA (Supplementary Table S3). No SNP pleiotropy was found in the level pleiotropy, according to the MR-Egger’s intercept term and the MR-PRESSO’s global test, demonstrating the strength of our instrumental variables (Table 2 and Supplementary Table S4). There were no potentially significant SNPs causing the causal relationship found by “leave-one-out” analysis using the IVW approach (Figure 4).

### Replication on the risk of smoking to OA

**Figure 5:**
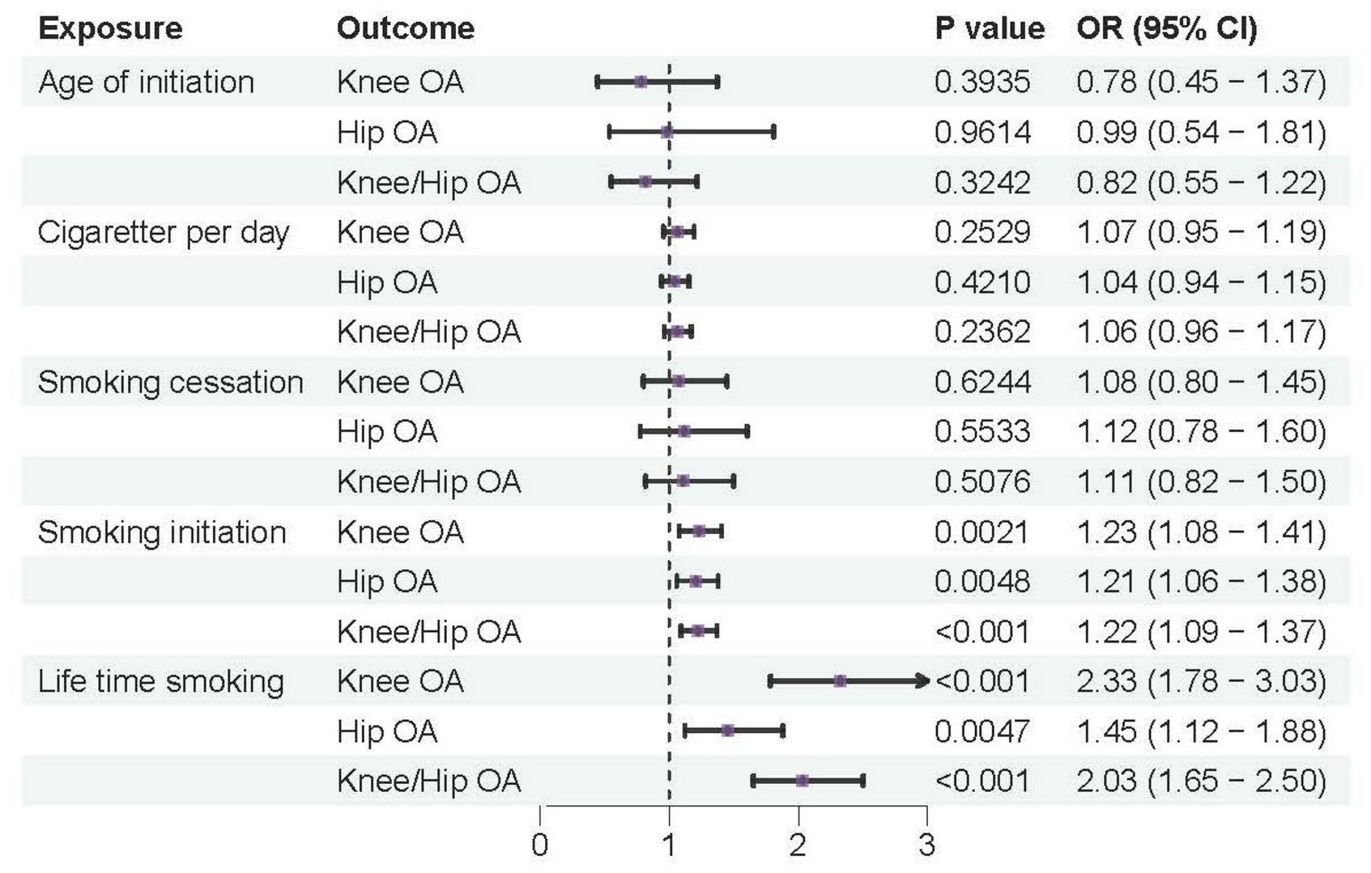
Forest plots of MR analysis by IVW for genetically predicted smoking phenotypes and risk of OA (replication).

According to the IVW analysis, a genetic predisposition to smoking initiation was linked to a higher risk of knee OA (OR = 1.23, CI = 1.08–1.41, FDR = 0.0081, *p* = 0.0021), hip OA (OR = 1.21, CI = 1.06–1.38, FDR = 0.0048, *p* = 0.0121) and knee/hip OA (OR = 1.22, CI = 1.09–1.37, FDR = 0.0026, *p* = 0.0005). Additionally, there was a significant positive correlation between genetically predicted lifetime smoking and knee OA (OR = 2.33, CI = 1.78–3.03, FDR < 0.001, *p* < 0.001), hip OA (OR = 1.45, CI = 1.12–1.88, FDR = 0.0142, *p* = 0.0047) and knee/hip OA (OR = 2.03, CI = 1.65–2.50, FDR < 0.001, *p* < 0.001) (Table 3 and Figure 5). Similarly with discovery analysis, the replication analysis also found a negative association between smoking cessation, age of initiation, cigarette per day and OA (Supplementary Table S6).

**Table 3:** Mendelian randomization results of causal links between smoking phenotypes and different OA phenotypes by using IVW method in replicate cohort.

Exposure	Outcome	OR (95% CI)	SE	*P*_IVW_	*P*_FDR_	Heterogeneity *P* for Cochran’s *Q*	*P* for MR-PRESSO	Horizontal pleiotropy *P* for Egger intercept
Age of initiation	Knee OA	0.78 (0.45–1.37)	0.2865	0.3935	0.5902	0.2514	0.3414	0.2040
	Hip OA	0.99 (0.54–1.81)	0.3099	0.9614	0.9614	0.7405	0.6492	0.5730
	Knee/Hip OA	0.82 (0.55–1.22)	0.2023	0.3242	0.5403	0.6568	0.3143	0.3190
Cigarette per day	Knee OA	1.07 (0.95–1.19)	0.0567	0.2529	0.4743	0.0003	0.0181	0.3120
	Hip OA	1.04 (0.94–1.15)	0.0514	0.4210	0.5741	0.1702	0.0344	0.2490
	Knee/Hip OA	1.06 (0.96–1.17)	0.0500	0.2362	0.5061	<0.001	0.0144	0.3040
Smoking cessation	Knee OA	1.08 (0.80–1.45)	0.1513	0.6244	0.669	0.1015	0.6132	0.0510
	Hip OA	1.12 (0.78–1.60)	0.1852	0.5533	0.6384	0.0557	0.4989	0.0810
	Knee/Hip OA	1.11 (0.82–1.50)	0.1546	0.5076	0.6346	0.0035	0.9338	0.1970
Smoking initiation	Knee OA	1.23 (1.08–1.41)	0.0677	0.0021	0.0081	<0.001	0.8727	0.8100
	Hip OA	1.21 (1.06–1.38)	0.0673	0.0048	0.0121	0.0412	0.6216	0.5800
	Knee/Hip OA	1.22 (1.09–1.37)	0.0581	0.0005	0.0026	<0.001	0.8028	0.7580
Life time smoking	Knee OA	2.33 (1.78–3.03)	0.1351	<0.001	<0.001	<0.001	0.0587	0.7410
	Hip OA	1.45 (1.12–1.88)	0.1320	0.0047	0.0142	0.0815	0.2660	0.7440
	Knee/Hip OA	2.03 (1.65–2.50)	0.1058	<0.001	<0.001	<0.001	0.0568	0.6280

All statistical tests were two-sided.

In the sensitivity analysis, evidence also existed suggesting that there was some heterogeneity in the MR analysis for the causal association of smoking with OA risk (Supplementary Table S7). There was no evidence of directional pleiotropy between smoking and OA risk, according to the MR-Egger intercept term and MR-PRESSO global test, suggesting a robust causal relationship (Table 3 and Supplementary Table S8). There were no potentially significant SNPs causing the causal relationship found by “leave-one-out” analysis using the IVW approach.

### Combined results from meta-analysis

**Figure 6:**
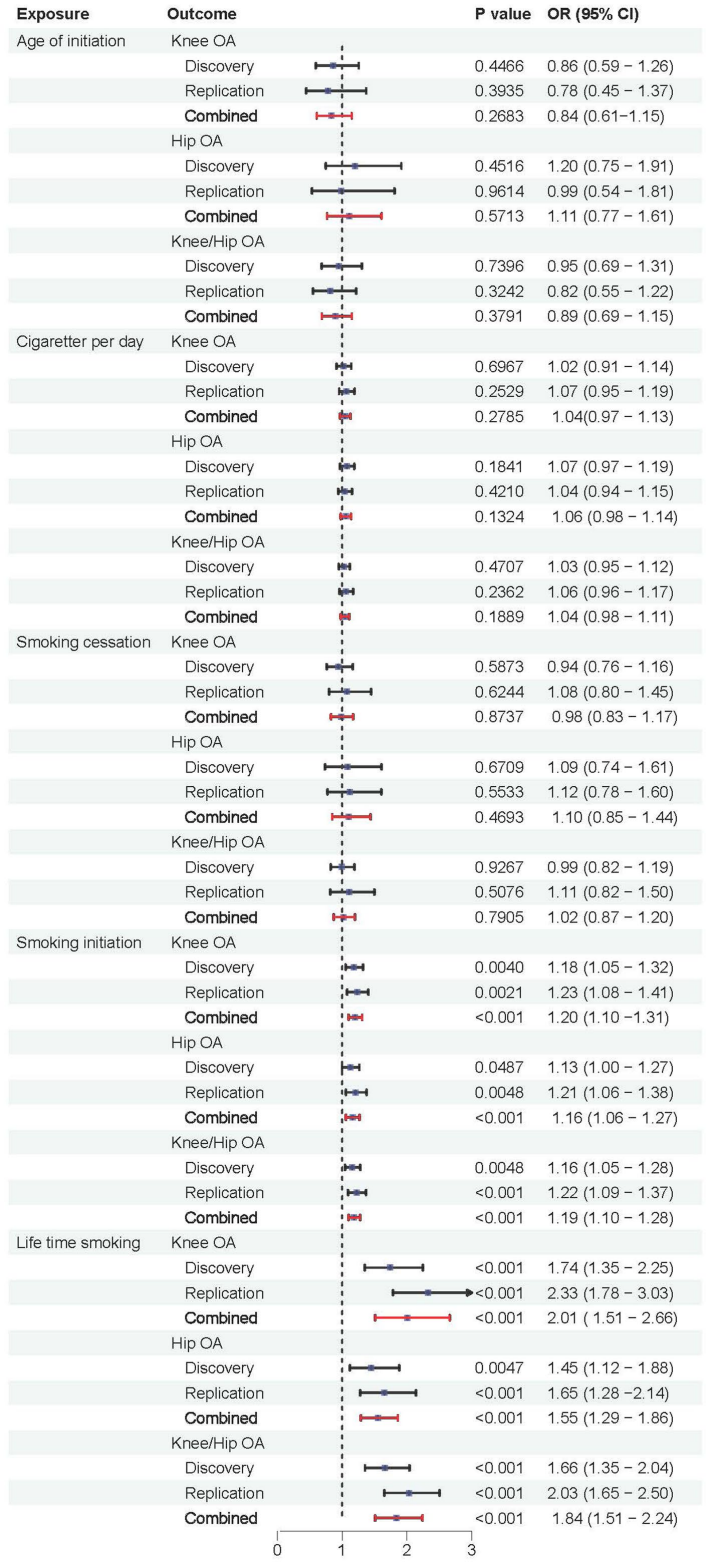
Forest plots of combined results from the meta-analysis of OA risk.

We further validated the positive causal relationship between smoking initiation, lifetime smoking and OA through meta-analysis (Figure 6). Smoking initiation was associated with an elevated risk of knee OA by 20%, hip OA by 16%, and knee/hip OA by 19% (all *p* < 0.001). Similarly, lifetime smoking was associated with a higher risk of knee OA by 101%, hip OA by 55%, and knee/hip OA by 84% (all *p* < 0.001). Meanwhile, age of initiation, cigarette per day and smoking cessation showed no significant causal relationships with OA.

## Discussion

Millions of individuals throughout the world suffer with OA, the most prevalent chronic degenerative joint disease and the primary source of pain and physical disability (22). This burden is further exacerbated by the rising demographic trends of obesity and aging within the population, which are putting additional strain on our healthcare system. The establishment of successful preventative plans and focused therapy interventions depends on the early identification and evaluation of OA risk factors. The effect of smoking as a modifiable risk factor on the occurrence and development of OA has yet to be conclusively clarified. We used the most extensive collection of smoking phenotype SNPs with identified sites and the largest OA GWAS database for discovery MR, along with an abundance of OA GWAS data for replication MR, to perform a thorough investigation into the causal relationships between smoking phenotypes and the risk of OA using a two-sample MR framework. Collectively, the integration of MR analysis and meta-analysis revealed a causal link between smoking initiation and lifetime smoking and the risk of OA.

The most extensive collection of smoking phenotypes to date, including age of smoking initiation, smoking initiation, cigarettes per day, smoking cessation, and lifetime smoking, was employed in this two-sample MR study. We discovered that lifetime smoking and smoking initiation were associated with a higher incidence of knee, hip, and knee/hip OA. The phenotype of smoking initiation was binary, representing either never or ever becoming a frequent smoker. Lifetime smoking is a continuous measure composite notion formed by smoking initiation, duration, heaviness, and quitting. It represents the burden of lifetime exposure to smoking. Compared to people who had never smoked, individuals who had ever smoked frequently were at a greater risk of developing OA. Furthermore, there was a significant correlation between an increased risk of OA and a 1-SD increase in the life time smoking index (i.e., smoking 20 cigarettes a day for 15 years and quitting 17 years ago, or smoking 60 cigarettes a day for 13 years and quitting 22 years ago). Prospective cohort research found that current smokers had a 2.20-fold greater risk of developing hand OA than non-smokers, which supports our findings on the independent negative effect of smoking (23). Likewise, a nationwide cross-sectional study carried out in the US has demonstrated a positive association between smoking and OA prevalence. In particular, current and previous smokers showed a higher risk of developing OA compared to non-smokers, with current smokers having a 54% higher risk and former smokers having a 38% higher risk (10).

As mentioned in the introduction, previous studies have produced mixed findings. In a cross-sectional and longitudinal investigation, there was no clear association between smoking and the incidence, prevalence, or advancement of hip OA (11). In this study, smoking status of participants was classified as current, former and never smoker, which is a qualitative classification that does not take into account factors such as the duration and heaviness of smoking. Another study that used microscopic analysis to examine the impact of smoking on meniscal tissues in OA knees found no differences between smokers’ and non-smokers’ menisci. The number of cigarettes smoked daily was also found to have a slight negative connection with the new Bonar score, indicating that the more cigarettes smoked daily, the less severe the negative involvement of meniscal histology (24). Hypothetically, this discovery could be attributed to the paradoxical benefit that transient ischemia induced by cigarette smoking might have on low-metabolism tissues such as the menisci.

The age at which a person starts smoking regularly is represented by the continuous phenotype known as “age of smoking initiation.” Early smoking is more harmful to health since tobacco smoke has a more noticeable neurotoxic effect (25). Since beginning to smoke before the age of 15 roughly doubles the risk of premature death in adulthood and beginning to smoke before the age of 10 nearly triples the risk of beginning to smoke at the age of 15 or older, the age of initiation in childhood and adolescence also appears to be significant (26). In over three quarters of the nations examined, the average age at which adolescents begin smoking cigarettes has either fallen or stayed the same, according to data from the most current Global Youth Tobacco Survey, which involved 99,728 teenagers (27). The results of the current study, which indicate a later age at which cigarette smoking initiation occurs, point to beneficial life cycle effects in lowering the total severity of smoking-related health consequences (28). Although no association was found between age of smoking and all type of OA in this study, considering that smoking has doubtless adverse effects on human health, smoking during early life should still be avoided.

The impact of OA on smoking behavior is complex and contradictory. Approximately 80% of the women with OA who smoked stopped during the three survey intervals, according to data from prospective cohort research. However, a tiny but noteworthy 10% of OA women who did not smoke also began smoking (29). Some women may have started smoking in order to assist manage their weight, or nicotine may have a pain-relieving effect. Given that smoking is a significant modifiable risk factor that can accelerate the development and progression of OA, stopping smoking may be a viable strategy for managing and preventing this debilitating joint condition. In our MR analysis, we discovered no correlation between smoking cessation and a lower incidence of OA. While quitting smoking may reduce systemic and local inflammation, thereby potentially inhibiting the onset or progression of OA, the concomitant weight gain often observed following smoking cessation could conversely elevate the risk of knee degenerative conditions (30).

The precise mechanism linking smoking to OA is currently unclear. Several mechanisms have been proposed to explain how smoking may lead to OA. One of the most prominent pathways involves the pro-inflammatory and catabolic effects of tobacco constituents, particularly nicotine. Nicotine has been demonstrated to trigger the release of pro-inflammatory cytokines from chondrocytes, including interleukin-1 (IL-1) and tumor necrosis factor-alpha (TNF-*α*), which in turn upregulate matrix metalloproteinases (MMPs) that degrade the cartilage extracellular matrix (31). Furthermore, smoking has been linked to decreased production of type II collagen and aggrecan, two essential articular cartilage constituents, upsetting the equilibrium between anabolic and catabolic processes in the joint (32). Moreover, oxidative stress resulting from the generation of ROS by cigarette smoking is another plausible mechanism linking smoking to OA. ROS can cause direct damage to chondrocytes and inhibit the production of essential matrix molecules, leading to cartilage degradation (33). The antioxidant defense system within the joint may be overwhelmed by the persistent oxidative challenge from smoking, contributing to a vicious cycle of inflammation and tissue damage (34). In addition to these molecular and cellular perturbations, the systemic effects of smoking, including vascular dysfunction (35), may further exacerbate joint pathology. The vasoconstrictive effects of nicotine can reduce blood flow to the joint, impairing nutrient supply and waste removal, while also hampering the delivery of immune cells and factors critical for tissue repair (36).

Although a number of MR investigations have examined the link between smoking and OA, to the best of our knowledge, their findings have not always been in agreement (14, 15, 37). With the largest sample size to date and the most comprehensive characterization of smoking phenotypes and outcomes, our MR study aims to provide valuable insights into the relationship between smoking and OA. Our study does still have certain limitations, though. First, heterogeneity was noted in this MR analysis. However, these heterogeneities had a minor impact on our analytical results since a random-effects model, specifically IVW was used, effectively diminished its influence on our results. Second, the majority of the data used for MR analysis came from European populations, which restricted the ability to extrapolate our findings across ethnic groups. Furthermore, because we used summary data, we were unable to perform a non-linear analysis to examine the threshold effect or a stratified analysis by smoking status.

## Conclusion

Overall, this MR analysis’s data clearly points to smoking’s etiological significance in the onset of OA. Additional research is required to identify the precise mechanism by which smoking contributes to OA. As the global burden of OA continues to rise, public health efforts aimed at reducing smoking prevalence remain crucial not only for overall health but also for the prevention of OA and other tobacco-related musculoskeletal disorders.

## Data Availability

The original contributions presented in the study are included in the article/Supplementary material, further inquiries can be directed to the corresponding author.

## References

[1] Boer CG Hatzikotoulas K Southam L Stefansdottir L Zhang Y Coutinho de Almeida R. Deciphering osteoarthritis genetics across 826,690 individuals from 9 populations. Cell. (2021) 184:6003–5. doi: 10.1016/j.cell.2021.11.003, PMID: 34822786 PMC8658458

[2] Brandt KD. Osteoarthritis. Preface. Rheum Dis Clin N Am. (2003) 29:9–13. doi: 10.1016/S0889-857X(03)00076-0, PMID: 14603575

[3] Cho Y Jeong S Kim H Kang D Lee J Kang SB. Disease-modifying therapeutic strategies in osteoarthritis: current status and future directions. Exp Mol Med. (2021) 53:1689–96. doi: 10.1038/s12276-021-00710-y, PMID: 34848838 PMC8640059

[4] Palazzo C Nguyen C Lefevre-Colau MM Rannou F Poiraudeau S. Risk factors and burden of osteoarthritis. Ann Phys Rehabil Med. (2016) 59:134–8. doi: 10.1016/j.rehab.2016.01.006, PMID: 26904959

[5] Nedunchezhiyan U Varughese I Sun AR Wu X Crawford R Prasadam I. Obesity, inflammation, and immune system in osteoarthritis. Front Immunol. (2022) 13:907750. doi: 10.3389/fimmu.2022.907750, PMID: 35860250 PMC9289681

[6] Watt FE. Posttraumatic osteoarthritis: what have we learned to advance osteoarthritis? Curr Opin Rheumatol. (2021) 33:74–83. doi: 10.1097/BOR.0000000000000760, PMID: 33186246

[7] Geng R Li J Yu C Zhang C Chen F Chen J. Knee osteoarthritis: current status and research progress in treatment (review). Exp Ther Med. (2023) 26:481. doi: 10.3892/etm.2023.12180, PMID: 37745043 PMC10515111

[8] Kwon HM Yang IH Park KK Cho BW Byun J Lee WS. Cigarette smoking and knee osteoarthritis in the elderly: data from the Korean National Health and nutritional examination survey. Exp Gerontol. (2020) 133:110873. doi: 10.1016/j.exger.2020.110873, PMID: 32044381

[9] Kong L Wang L Meng F Cao J Shen Y. Association between smoking and risk of knee osteoarthritis: a systematic review and meta-analysis. Osteoarthr Cartil. (2017) 25:809–16. doi: 10.1016/j.joca.2016.12.020, PMID: 28011100

[10] Zhu S Ji L He Z Zhang W Tong Y Luo J. Association of smoking and osteoarthritis in US (NHANES 1999-2018). Sci Rep. (2023) 13:3911. doi: 10.1038/s41598-023-30644-6, PMID: 36890196 PMC9995311

[11] Salis Z. Investigation of the associations of smoking with hip osteoarthritis: a baseline cross-sectional and four- to five-year longitudinal multicohort study. ACR Open Rheumatol. (2024) 6:155–66. doi: 10.1002/acr2.11644, PMID: 38174808 PMC10933634

[12] Bowden J Holmes MV. Meta-analysis and Mendelian randomization: a review. Res Synth Methods. (2019) 10:486–96. doi: 10.1002/jrsm.1346, PMID: 30861319 PMC6973275

[13] Ni J Wang P Yin KJ Huang JX Tian T Cen H. Does smoking protect against developing osteoarthritis? Evidence from a genetically informed perspective. Semin Arthritis Rheum. (2022) 55:152013. doi: 10.1016/j.semarthrit.2022.152013, PMID: 35500427

[14] Lee YH. Causal association between smoking behavior and the decreased risk of osteoarthritis: a Mendelian randomization. Z Rheumatol. (2019) 78:461–6. doi: 10.1007/s00393-018-0505-7, PMID: 29974223

[15] Johnsen MB Vie GA Winsvold BS Bjorngaard JH Asvold BO Gabrielsen ME. The causal role of smoking on the risk of hip or knee replacement due to primary osteoarthritis: a Mendelian randomisation analysis of the HUNT study. Osteoarthr Cartil. (2017) 25:817–23. doi: 10.1016/j.joca.2016.12.021, PMID: 28049019

[16] Skrivankova VW Richmond RC Woolf BAR Yarmolinsky J Davies NM Swanson SA. Strengthening the reporting of observational studies in epidemiology using Mendelian randomization: the STROBE-MR statement. JAMA. (2021) 326:1614–21. doi: 10.1001/jama.2021.18236, PMID: 34698778

[17] Liu M Jiang Y Wedow R Li Y Brazel DM Chen F. Association studies of up to 1.2 million individuals yield new insights into the genetic etiology of tobacco and alcohol use. Nat Genet. (2019) 51:237–44. doi: 10.1038/s41588-018-0307-5, PMID: 30643251 PMC6358542

[18] Wootton RE Richmond RC Stuijfzand BG Lawn RB Sallis HM Taylor GMJ. Evidence for causal effects of lifetime smoking on risk for depression and schizophrenia: a Mendelian randomisation study. Psychol Med. (2020) 50:2435–43. doi: 10.1017/S0033291719002678, PMID: 31689377 PMC7610182

[19] Zengini E Hatzikotoulas K Tachmazidou I Steinberg J Hartwig FP Southam L. Genome-wide analyses using UK biobank data provide insights into the genetic architecture of osteoarthritis. Nat Genet. (2018) 50:549–58. doi: 10.1038/s41588-018-0079-y, PMID: 29559693 PMC5896734

[20] Bowden J Del Greco MF Minelli C Davey Smith G Sheehan NA Thompson JR. Assessing the suitability of summary data for two-sample Mendelian randomization analyses using MR-Egger regression: the role of the I2 statistic. Int J Epidemiol. (2016) 45:1961–74. doi: 10.1093/ije/dyw220, PMID: 27616674 PMC5446088

[21] Burgess S Bowden J Fall T Ingelsson E Thompson SG. Sensitivity analyses for robust causal inference from Mendelian randomization analyses with multiple genetic variants. Epidemiology. (2017) 28:30–42. doi: 10.1097/EDE.0000000000000559, PMID: 27749700 PMC5133381

[22] He Y Li Z Alexander PG Ocasio-Nieves BD Yocum L Lin H. Pathogenesis of osteoarthritis: risk factors, regulatory pathways in chondrocytes, and experimental models. Biology. (2020) 9:194. doi: 10.3390/biology9080194, PMID: 32751156 PMC7464998

[23] Haugen IK Magnusson K Turkiewicz A Englund M. The prevalence, incidence, and progression of hand osteoarthritis in relation to body mass index, smoking, and alcohol consumption. J Rheumatol. (2017) 44:1402–9. doi: 10.3899/jrheum.170026, PMID: 28711879 PMC5878837

[24] Zabrzynska M Pasinski M Gagat M Kulakowski M Wozniak L Elster K. The association between the extent of the osteoarthritic Meniscus degeneration and cigarette smoking-a pilot study. Medicina. (2024) 60:323. doi: 10.3390/medicina60020323 38399610 PMC10890507

[25] DeBry SC Tiffany ST. Tobacco-induced neurotoxicity of adolescent cognitive development (TINACD): a proposed model for the development of impulsivity in nicotine dependence. Nicotine Tob Res. (2008) 10:11–25. doi: 10.1080/14622200701767811, PMID: 18188741

[26] Thomson B Rojas NA Lacey B Burrett JA Varona-Perez P Martinez MC. Association of childhood smoking and adult mortality: prospective study of 120 000 Cuban adults. Lancet Glob Health. (2020) 8:e850–7. doi: 10.1016/S2214-109X(20)30221-7, PMID: 32446350 PMC7248573

[27] Xing S Zhao M Magnussen CG Xi B. Proportion and trend in the age of cigarette smoking initiation among adolescent smoking experiencers aged 13-15 years in 148 countries/territories. Front Public Health. (2022) 10:1054842. doi: 10.3389/fpubh.2022.1054842, PMID: 36518585 PMC9742527

[28] National Center for Chronic Disease Prevention and Health Promotion (US) Office on Smoking and Health. The health consequences of Smoking-50 years of Progress: a report of the surgeon general. Atlanta (GA): Reports of the Surgeon General (2014).

[29] Ng N Parkinson L Brown WJ Moorin R Peeters G. Lifestyle behaviour changes associated with osteoarthritis: a prospective cohort study. Sci Rep. (2024) 14:6242. doi: 10.1038/s41598-024-54810-6, PMID: 38485979 PMC10940587

[30] Zeng C Nguyen UDT Wu J Wei J Luo X Hu S. Does smoking cessation increase risk of knee replacement? A general population-based cohort study. Osteoarthr Cartil. (2021) 29:697–706. doi: 10.1016/j.joca.2021.02.382, PMID: 33621706

[31] Yang X Qi Y Avercenc-Leger L Vincourt JB Hupont S Huselstein C. Effect of nicotine on the proliferation and chondrogenic differentiation of the human Wharton's jelly mesenchymal stem cells. Biomed Mater Eng. (2017) 28:S217–28. doi: 10.3233/BME-171644, PMID: 28372298

[32] Heluany CS Kupa LVK Viana MN Fernandes CM Silveira ELV Farsky SHP. In vivo exposure to hydroquinone during the early phase of collagen-induced arthritis aggravates the disease. Toxicology. (2018) 408:22–30. doi: 10.1016/j.tox.2018.06.010, PMID: 29935983

[33] Kumar SN Bastia B Borgohain D Agrawal U Raisuddin S Jain AK. Structural changes, increased hypoxia, and oxidative DNA damage in placenta due to maternal smokeless tobacco use. Birth Defects Res. (2021) 113:1198–214. doi: 10.1002/bdr2.1941, PMID: 34288583

[34] Fernandez-Torres J Aztatzi-Aguilar OG Zamudio-Cuevas Y Sierra-Vargas MP Martinez-Nava GA Montano-Armendariz N. Effect of smoking on the redox status of knee osteoarthritis: a preliminary study. Exp Biol Med. (2023) 248:1754–67. doi: 10.1177/15353702231199072, PMID: 37916410 PMC10792422

[35] Bazzano LA He J Muntner P Vupputuri S Whelton PK. Relationship between cigarette smoking and novel risk factors for cardiovascular disease in the United States. Ann Intern Med. (2003) 138:891–7. doi: 10.7326/0003-4819-138-11-200306030-00010, PMID: 12779299

[36] McDaniel JC Browning KK. Smoking, chronic wound healing, and implications for evidence-based practice. J Wound Ostomy Continence Nurs. (2014) 41:415–23. doi: 10.1097/WON.0000000000000057, PMID: 25188797 PMC4241583

[37] Larsson SC Burgess S. Appraising the causal role of smoking in multiple diseases: a systematic review and meta-analysis of Mendelian randomization studies. EBioMedicine. (2022) 82:104154. doi: 10.1016/j.ebiom.2022.104154, PMID: 35816897 PMC9278068

