# Supplementary material for: The causal impact of smoking behavior on osteoarthritis: a Mendelian randomization analysis
Source: Front Public Health. 2025 Jan 23;13:1437443. doi: 10.3389/fpubh.2025.1437443 (PMC11798882; doi:10.3389/fpubh.2025.1437443)
Supplement: Supplementary file 2 [file Table_1.docx]

Supplementary Material

Supplementary Tables

Supplementary Table 1. Discovery extracting Information from SNPs.

Supplementary Table 2. Discovery positive MR analysis results.

Supplementary Table 3. Discovery heterogeneity test.

Supplementary Table 4. Discovery level pleiotropy and PRESSO test.

Supplementary Table 5. Replication extracting Information from SNPs.

Supplementary Table 6. Replication positive MR analysis results.

Supplementary Table 7. Replication heterogeneity test.

Supplementary Table 8. Replication level pleiotropy and PRESSO test.
